# Double primary hepatic cancer (hepatocellular carcinoma and intrahepatic cholangiocarcinoma) originating from hepatic progenitor cell: a case report and review of the literature

**DOI:** 10.1186/s12957-016-0974-6

**Published:** 2016-08-17

**Authors:** Junwen Hu, Rongfa Yuan, Changwen Huang, Jianghua Shao, Shubing Zou, Kai Wang

**Affiliations:** Second Affiliated Hospital of Nanchang University, No.1, Minde Road, Nanchang, 330006 China

**Keywords:** Hepatocellular carcinoma, Intrahepatic cholangiocarcinoma, Double hepatic cancer, Hepatic progenitor cell, Prognosis

## Abstract

**Background:**

Synchronous development of primary hepatocellular carcinoma (HCC) and intrahepatic cholangiocarcinoma (ICC) in different sites of the liver have rarely been reported before. The purpose of this study is to investigate the clinicopathological characteristics of synchronous double cancer of HCC and ICC.

**Case presentation:**

A 56-year-old Chinese man without obvious liver cirrhosis was preoperation diagnosed with multiple HCC in segments VI (SVI) and VII (SVII) by the abdominal computed tomography (CT) and contrast-enhanced ultrasonography (CEUS). We performed hepatic resection of both segments. The tumors in SVI and SVII were pathologically diagnosed as ICC and HCC, respectively. Immunohistochemically, the HCC in SVII was positive for HepPar-1 and negative for CK19, while the ICC in SVI tumor was positive for CK19 and negative for HepPar-1. Interestingly, the immunohistochemical results also showed that the classic hepatic progenitor cell (HPCs) markers CD34 and CD117 were both positive of the two tumors. The patient still survived and at a 1-year follow-up did not show evidence of metastasis or new recurrent lesions. We speculate that the two masses may have originated from HPCs based on the findings of this patient.

**Conclusions:**

Synchronous development of HCC and ICC is very rare with unique clinical and pathological features. The correct preoperative diagnosis of double hepatic cancer of HCC and ICC is difficult. Hepatitis B virus (HBV) and hepatitis C virus (HCV) infection were both the independent risk factor to the development of double liver cancer. Hepatic resection is the preferred and most effective treatment choice. The prognosis of synchronous occurrence of double hepatic cancer was poorer than for either HCC or ICC, and the origin of it needs further study.

## Background

Primary liver cancers are divided into hepatocellular carcinoma (HCC), intrahepatic cholangiocarcinoma (ICC), and combined hepatocellular carcinoma and cholangiocarcinoma (cHCC-CC) according to the histological type. HCC and ICC are the two most common primary liver cancers. There are reports of cancers with cHCC-CC in a single tumor [1, 2]; however, double primary liver cancer comprising different primary hepatic tumors in the same liver is exceedingly rare [3–5]. We herein present a patient who underwent hepatic resection for double primary hepatic cancer without a background of obvious liver cirrhosis. We also review the current literature of related clinicopathological features and investigate the origin of double primary hepatic cancer.

## Case presentation

**Fig. 1:**
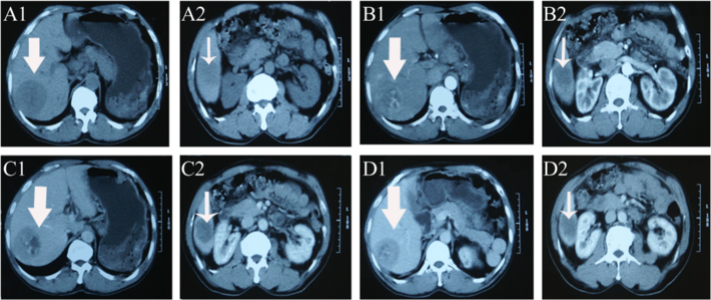
Computed tomography (CT) imaging of the patient with a double hepatic cancer of hepatocellular carcinoma and intrahepatic cholangiocarcinoma. Abdominal unenhanced CT showed two separate low-density tumors, in SVII (**A1**, *large arrow*) and SVI (**A2**, *small arrow*) of the liver. On contrast-enhanced dynamic CT, the SVII (**B1**, *large arrow*) tumor was enhanced, and the SVI (**B2**, *small arrow*) tumor showed peripheral enhancement during the arterial phase. Followed by wash-out during the portal venous phase (**C1**, **C2**) and the delayed phase (**D1**, **D2**): during the portal venous phase, the SVII (**C1**, *large arrow*) and SVI (**C2**, *small arrow*) tumors showed negative enhancement, and the density of SVII tumor rapidly decreased. During the delayed phase, the density of SVII (**D1**, *large arrow*) tumor further decreased, and the SVI (**D2**, *small arrow*) tumor showed slightly centripetal enhancement

**Fig. 2:**
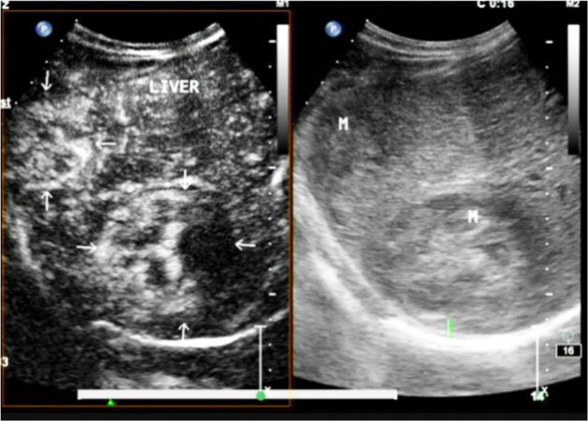
There were different CEUS finding features in two separate liver lesions

**Fig. 3:**
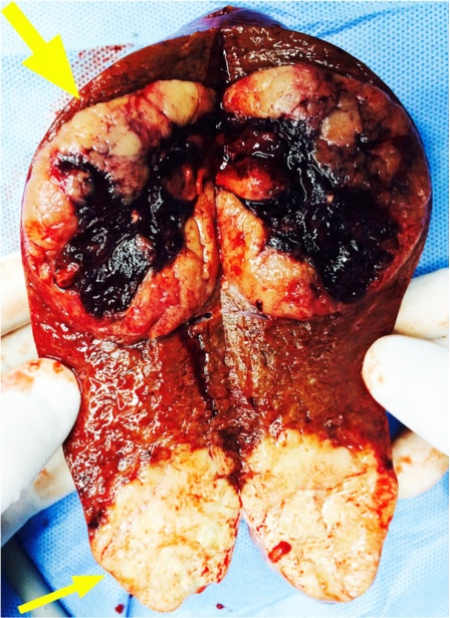
The cut surface of the resected specimen showed two tumors, one in SVII (*upper arrow*) is HCC and one in SVI (*lower arrow*) is ICC

**Fig. 4:**
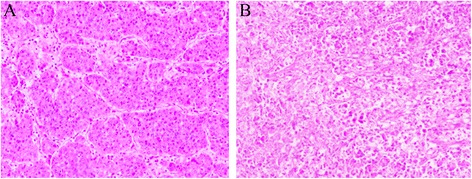
Histological findings of the liver tumors (hematoxylin-eosin staining, ×100). **a** The SVII tumor was pathologically diagnosed as a moderately differentiated hepatocellular carcinoma (HCC) (with nodular, trabecular). **b** The SVI tumor was pathologically diagnosed as a poorly differentiated cholangiocellular carcinoma (ICC) (with mucin-producing glands)

**Fig. 5:**
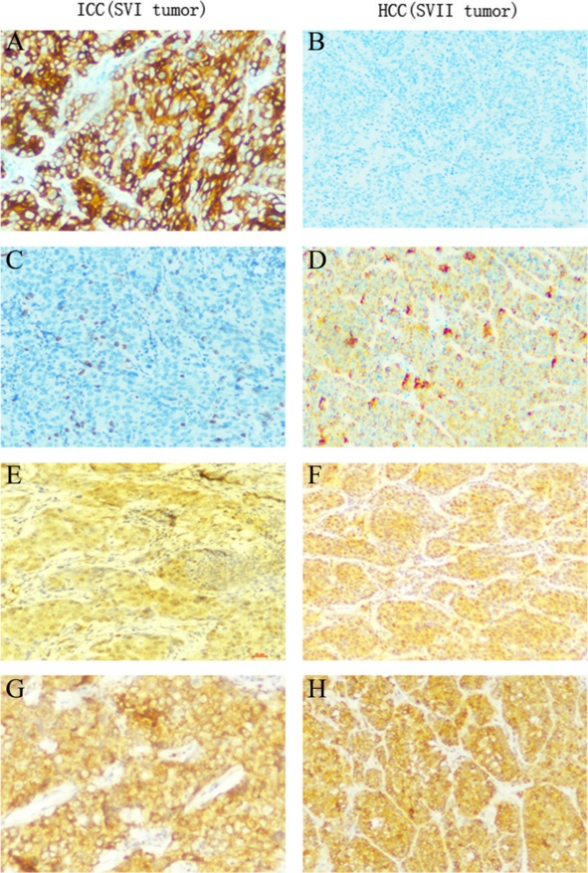
**a**, **b** Immunohistochemical staining for CK19. This was positive in the SVI tumor (ICC) **a** and negative in the SVII tumor(HCC). **b c**, **d** Immunohistochemical staining for HepPar I. This was negative in the SVI tumor **c** and positive in the SVII tumor. **d e**, **f** Immunohistochemical staining for hepatic progenitor cell marker CD34. The SVI and SVII tumor were both positive for CD34. **g**, **h** Immunohistochemical staining for hepatic progenitor cell marker CD117. The SVI and SVII tumor were both positive for CD117. **a**–**h** ×100

A 56-year-old man was admitted to our hospital with the chief complaint of lumbago in the right side for 1 week. He had a history of splenectomy and blood transfusions due to spleen rupture. However, he had no history of exposure to any hepatotoxic chemicals and no family history of liver disease. Physical examination findings upon admission were normal. Laboratory examination produced the following results: white blood cell count, 6.61 × 10^9^/l; red blood cell count, 3.70 × 10^12^/l; hemoglobin, 120 g/l; albumin, 39.811 g/l; globulin, 26.81 g/l; total bilirubin, 9.59 μmol/l; and prothrombin time, 14.9 s. The alanine aminotransferase, aspartate aminotransferase, and lactate dehydrogenase levels were within normal ranges. Hepatitis B virus-related antigen and antibody were negative with the exception of hepatitis B e antibody (HBeAb) and hepatitis B core antibody (HBcAb). Anti-HCV antibody was negative. The carbohydrate antigen 19-9, α-fetoprotein, and carcinoembryonic antigen levels were 166.77 U/ml, 7.0 ng/ml, and 0.98 ng/ml, respectively. Preoperative abdominal computed tomography (CT) and contrast-enhanced ultrasonography (CEUS) provided vivid images of two separate lesions with different contrast features in segment VI (SVI, 4.5 × 4 cm in size) and segment VII (SVII, 6 × 5 cm in size) (Figs. 1 and 2), suggesting tumors of different origins. The patient was diagnosed with double primary hepatic cancer (hepatocellular carcinoma and intrahepatic cholangiocarcinoma) and underwent partial liver hepatectomy and cholecystectomy. The resected specimen contained a soft, red, focal hemorrhage tumor (7 × 5.5 × 5.5 cm in size) in SVII, and a hard, off-white tumor (4.5 × 4 × 4 cm in size) in SVI (Fig. 3). The SVII tumor was histologically confirmed as a typical moderately differentiated HCC with a trabecular pattern. The SVI tumor was pathologically diagnosed as a poorly differentiated ICC with mucin-producing glands (Fig. 4). Immunohistochemically, the SVII tumor was positive for HepPar-1 and negative for CK19, and the SVI tumor was positive for CK19 and negative for HepPar-1. We further examined the HPCs markers immunohistochemically, and the results showed that the classic HPCs markers CD34 and CD117 were both positive of the two tumors (Fig. 5). The hepatic tissue adjacent to the tumors showed no liver cirrhosis. According to the histological findings, the liver tumors in this patient were diagnosed as a double primary liver cancer. The postoperative course was uneventful, and he was discharged on the seventh postoperative day. The patient survived and at 1-year follow-up did not show evidence of metastasis or new recurrent lesions.

### Discussion

Combined hepatocellular carcinoma and cholangiocarcinoma (cHCC-CC) is a rare primary liver tumor (PLC) that contains both hepatocellular carcinoma (HCC) and cholangiocarcinoma (ICC) and only accounts for 0.4 to 14.2 % of PLCs [6]. Although Wells reported cHCC-CC in 1903, the pathological histology, biological behavior, clinical characteristics, and prognosis of cHCC-CC are still lacking compared with those HCC and ICC [7–9]. Currently, there is no uniform pathology classification of cHCC-CC, and there are mainly two types of classification. Allen and Lisa [10] first divided cHCC-CC into three types: type A (HCC and ICC grow independently in different parts of the same liver, but have clear boundaries); type B (HCC and ICC originate from different cells and mingle as they grow; however, there are still certain boundaries between the two populations); and type C (HCC and ICC are completely integrated within the same tumor). Type C tumors are widely accepted as true cHCC-CC [11]. However, Goodman [12] put forward another pathological classification system that differed from that of Allen in 1985 and included type I (collision), type II (transitional), and type III (fibrolamellar) tumors. Based on the classification, we defined the liver tumors in our patient as type A or type I.

The cellular origin of double primary hepatic cancer is still unclear. Several studies have been proposed for such tumors. Some reports have shown that the cells which have already differentiated into both hepatocytes and choangiocytes before malignant transformation. Others revealed that activated HPCs were considered to be involved in carcinogenesis; HPCs have the potential to differentiate into either hepatocytes or cholangiocytes [4].

HPCs are thought to reside in the canals of Hering and can be activated and contribute to liver self-renewal and repair in response to liver injury by proliferating and differentiating towards both hepatocytes and biliary epithelial cells [13]. HPCs play an important role in the development and prognosis of the liver cancer [14–18]. There are a great number of studies revealing that HPCs are one probable origin of liver cancer. Reya [19] suggested that there is an affinity between HPCs and primary liver cancer according to the hypothesis of cancer stem cells and biological behaviors of HPCs. The fact that many liver tumors contain a mixture of mature cells and cells phenotypically similar to HPCs is the proof that HPCs may be possible to give rise to liver cancers [20]. HPCs are long-life cells that they are competent of surviving long enough to store up DNA damages, and the mutations are shared by lots of inferiors of the mutant progenitor cells; they become tumors more easily than other cell types [21, 22]. Research shows that HPCs contain the potential to differentiate into either hepatocytes or cholangiocytes, which also suggests that liver tumors may originate from HPCs [23]. HPCs have a powerful self-renewal capacity and rapid proliferation potential when the adult hepatocytes are heavily and continuously impaired beyond the ability to self-repair which dramatically increases the risk of progenitor cell mutations and is an indispensable approach for malignant transformation [24].

HPCs markers are used to identify the HPCs; there are a larger number of HPCs markers, and CD34 and CD117 are two of the classic human HPCs markers [25, 26]. We performed the immunohistochemical analysis of CD34 and CD117 to examine whether the two tumors arose from the HPCs in our study. The result showed that CD34 and CD117 were both positive of the two tumors, so we conclude that the two nodules composing the double primary hepatic cancer herein reported may originated from the hepatic progenitor cell.

Double hepatic cancer of HCC and ICC is rarely encountered in clinical practice, and according to the retrospective analysis of Watanable [27], the incidence of double primary liver cancer was 0.54–0.70 % of PLCs, and the male to female ratio of double cancer was 10:1. The average age of double cancer patients was 67, the rates of HBV and HCV infection were 9.1 and 72.7 %, respectively, and cirrhosis was observed in 36.4 % of patients. The rates of patients positive for expression of AFP and CA19-9 were 75.8 and 57.1 %, respectively. In contrast, Cao [28] retrospectively summarized the clinical data of 35 patients with simultaneous double cancer of HCC and ICC in his study, and the results showed that the incidence of double hepatic cancer was 0.25 %, the male to female ratio was 4:1, the average age of patients was 50 years old, the HBV and HCV infection rates were 100 and 60 %, respectively, all of the patients had cirrhosis, and simultaneously elevated AFP and CA19-9 levels were observed in 28.6 % of patients. It has been suggested that the background of double cancer is distinctly different from that of the cHCC-CC. In our study, the 56-year-old male had a history of HBV infection; however, this was not associated with obvious cirrhosis of the liver, and his AFP and CA19-9 levels were normal upon serological examination.

Surgical resection remains the preferred and most effective treatment option for double primary liver cancer and is especially suitable for patients without cirrhosis of the liver [29, 30]. However, commonly combined liver cirrhosis has the risk of serious complications, so strict choice of patients before surgery is important, and general physical condition should be considered, for example, any pre-existing cirrhosis and tumor extent [31, 32]. Recently, aggressive treatments including liver transplantation have been used on patients as a radical approach [33]; however, the long-term curative effect of liver transplantation need to be further studied. Transcatheter arterial chemoembolization (TACE), percutaneous ethanol injection (PEI) and radiofrequency ablation (RFA) are widely used for unresectable HCC and patients with recurrence after resection. However, the double hepatic tumor contains more fibrous tissue and fewer vascular components than HCC, and therefore, the therapeutic effect of TACE or PEI is limited [34]. In our case, we performed hepatic resection on the patient. The patient survived and at 1-year follow-up did not show evidence of metastasis or new recurrent lesions.

The prognosis of double primary cancer varies among different studies. However, the prognosis of double primary cancer has commonly been recognized to be poorer than for either HCC or ICC due to metastasis [30]. Cao retrospectively studied the clinical characteristics of 35 cases of double primary cancer and revealed that tumor recurrence developed in 77.1 % of patients, and distant metastases were detected in 20 % of patients after partial hepatectomy. The median overall survival (OS) was 18 months, and the 1-year, 3-year, 5-year OS rates were 60.0, 28.9, and 23.1 %, respectively [28].

## Conclusions

In summary, we have reported a rare case of double primary liver cancer verified by pathological and immunohistochemical analysis. The clinical information remains inconclusive because of its rarity. There are many aspects of double primary liver cancer that differ from HCC or ICC. The preoperatively diagnosis is more difficult, the therapeutic effect remains unclear, the origin of it need further study, and the prognosis is poorer. We need to pay more attention to double primary liver cancer, and we need to accumulate more cases for further research.

### Consent

Consent was obtained from the patient for publication of this case report.

## Abbreviations

CEUS, contrast-enhanced ultrasonography; cHCC-CC, combined hepatocellular carcinoma and cholangiocarcinoma; CT, computed tomography; HCC, primary hepatocellular carcinoma; HPCs, hepatic progenitor cells; ICC, intrahepatic cholangiocarcinoma; PEI, percutaneous ethanol injection; PLC, primary liver tumor; RFA, radiofrequency ablation; TACE, Transcatheter arterial chemoembolization
